# Pasteurized *Akkermansia muciniphila* improves glucose metabolism is linked with increased hypothalamic nitric oxide release^☆^

**DOI:** 10.1016/j.heliyon.2023.e18196

**Published:** 2023-07-13

**Authors:** Anne Abot, Amandine Brochot, Nicolas Pomié, Gwendoline Astre, Céline Druart, Willem M. de Vos, Claude Knauf, Patrice D. Cani

**Affiliations:** a Enterosys SAS, 31670, Labège, France; b The Akkermansia Company, 1435, Mont-Saint-Guibert, Belgium; c Laboratory of Microbiology, Wageningen University, 6700, EH Wageningen, the Netherlands; d Human Microbiome Research Program, University of Helsinki, 00014 Helsinki, Finland; e INSERM U1220, Institut de Recherche en Santé Digestive (IRSD), Université Paul Sabatier, Toulouse III, CHU Purpan, Place du Docteur Baylac, CS, 60039, CEDEX 3, 31024, Toulouse, France; f NeuroMicrobiota, International Research Program (IRP) INSERM/UCLouvain, France; g Metabolism and Nutrition Research Group, Louvain Drug Research Institute (LDRI), UCLouvain, Université Catholique de Louvain, Brussels, Belgium; h WELBIO-Walloon Excellence in Life Sciences and Biotechnology, WELBIO department, WEL Research Institute, Avenue Pasteur, 6, 1300, Wavre, Belgium

**Keywords:** Paraprobiotics, Enteric nervous system, Gut-brain axis, Diabetes

## Abstract

**Background and objective:**

Pasteurized *Akkermansia muciniphila* cells have shown anti-diabetic effects in rodents and human. Although, its primary site of action consists in maintaining the gut barrier function, there are no study exploring if *A. muciniphila* controls glycemia *via* a gut to brain axis. Targeting the gut motility represents an alternative pathway to treat hyperglycemia. Here, we tested the impact of pasteurized *A. muciniphila* on gut motility, gut-brain axis and glucose metabolism.

**Methods:**

We used mice fed a 45% high-fat (HFD) treated or not with pasteurized *A. muciniphila* Muc^T^ during 12 weeks. We measured the effects of the treatment on body weight gain, glucose metabolism (insulin, glycemia, glucose tolerance), gut contraction and enteric neurotransmitter release, and hypothalamic nitric oxide (NO) release.

**Results:**

We show that pasteurized *A. muciniphila* exerts positive effects on different metabolic parameters such as body weight, fat mass, insulin, glycemia and glucose tolerance. This could be explained by the ability of pasteurized *A. muciniphila* supplementation to decrease duodenal contraction and to increase hypothalamic NO release in HFD mice.

**Conclusion:**

We demonstrate a novel mode of action of pasteurized *A. muciniphila* explaining its beneficial impact on the control of glycemia in a preclinical model of type 2 diabetes *via* gut-brain axis signaling.

## 1 Introduction

The control of glycemia requires a fine-tuned crosstalk occurring between all organs and thanks to hormonal and nervous signals. Among all type of communications, the gut-brain axis is one of the most important physiological systems implicated in the maintenance of a normal glycemia [1]. Indeed, nutrients such as glucose could be detected by specialized cells in the gut including enterocytes and/or enteric neurons [2] thereby leading to changes in the release of hypothalamic neurotransmitters [3]. In response to intestinal glucose detection, the hypothalamus sends a message to metabolic tissues (liver, skeletal muscle) to modify glucose entry [4,5]. In this physiological context, intestinal glucose detection leads to an increase of nitric oxide (NO) release from the hypothalamus [3]. This phenomenon is associated with an increase of glucose uptake by tissues [6]. At the opposite, the alteration of glucose detection observed in a preclinical model of type 2 diabetes (T2D) is correlated with a dysfunction of hypothalamic NO release which contributed to insulin resistance [3].

Recently, we have discovered a new concept showing the implication of the enteric nervous system (ENS) in the control of glycemia *via* the gut-brain axis (GBA) [1]. ENS is notably composed by glial cells, interstitial Cajal cells and enteric neurons that work together as a syncytium to control intestinal motility from the duodenum to the colon. In physiological fed conditions, there is a positive correlation between the increase of duodenal contraction and glucose absorption [7]. We have shown that apelin, a bioactive peptide released by enterocytes in response to glucose [8], stimulates the release of acetylcholine by enteric neurons, a neurotransmitter that provokes gut contraction. This modification of gut motility is associated with a decrease of hypothalamic NO release that prevents glucose entry in muscles [8]. From a physiological point of view, this phenomenon appears at the very beginning of food intake with the aim to increase glucose absorption and eventually preventing hypoglycemia. Both type 2 diabetic patients and diabetic mice fed a high-fat diet present an alteration of ENS function. The expression of Choline Acetyl-Transferase (ChAT) and neuronal Nitric Oxide Synthase (nNOS) enzymes are respectively increased and decreased in the duodenum in diabetes [9]. These two enzymes generate two neurotransmitters that target the intestinal smooth muscle cells that is acetylcholine (from ChAT), the principal stimulatory neurotransmitter; and NO (from nNOS), the principal inhibitory neurotransmitter. In this pathological context, the modification of the ratio ChAT/nNOS observed in the duodenum is associated with a duodenal hypermotility that altered the GBA and hypothalamic NO release to favor insulin-resistance [8,10]. Therefore, targeting the couple “ENS/smooth muscle cells” in order to decrease the duodenal contraction, and then modify this mechano-detection system to restore the hypothalamic NO release represents an innovative pathway to treat T2D [1].

Several molecules named enterosynes [1] originating from the gut have the capacity to modulate duodenal contraction by targeting the ENS. Enterosynes are chemically diverse and related to hormones, bioactive peptides/lipids, nutrients, microbiota and immune factors [10]. In addition to this pharmacological action, we have shown that modulating the gut microbiota by using specific prebiotics (i.e. oligofructose) modifies duodenal motility and correlates with an improvement of diabetic state [11]. Hence, discovering bacteria or bacterial components that could exert a beneficial action on the couple “ENS/smooth muscle cells” may bring alternative and/or complementary approach to pharmacological treatments for T2D.

During the last 10 years, researchers have focused their attention on *A. muciniphila*, a gut bacterium with positive actions on glucose homeostasis [[12], [13], [14]]. This bacterium has the advantage to exert its action on glycemia in its alive form, but also in its pasteurized form conferring it paraprobiotic-type beneficial properties in the body [13,15,16]. Given the lack of data on the effect of this bacterium on ENS function associated metabolic disorders, we aimed to study the effect of pasteurized *A. muciniphila* on glucose absorption and on the GBA axis modulation by enteric neurons.

## 2 Methods

### 2.1 Production of pasteurized *A. muciniphila*

The production of the bacteria follows good manufacturing practice (GMP) and hazard analysis critical control points (HACCP) principles. In brief, the production process involves the successive preculture of *A. muciniphila* in liquid media under strictly anaerobic conditions. The inoculum is gradually increased in volume to prepare for the main fermenter. The main fermentation takes place under anaerobic conditions. The growth of *A. muciniphila* is monitored using spectrophotometry at regular intervals. The culture medium was cultured anaerobically in a synthetic medium where mucin was replaced by soy-peptone, threonine, and a mix of glucose and N-acetylglucosamine. The production process for pasteurized *A. muciniphila* includes anaerobic fermentation, followed by pasteurization and concentration of the bacterial cells. The cells are then mixed with cryoprotectants and freeze-dried to produce a powder. Total bacterial cell counting is performed, and stabilizing agents may be added if needed. The addition of stabilizing agents to dilute the powder to a specific cell count based on production yield and cell quantification in the raw powder. Currently, the bacteria were standardized at 10^11^ cells (measured as total fluorescent units (TFU)) per gram of powder. The powder is packaged in multi-layer pouches that are water- and airproof, heat sealed and stored at ≤ −18 °C until utilization. Pasteurization consisted of heat treatment at 70 °C for 30 min of fresh *A. muciniphila*.

### 2.2 Animals

Nine-week-old male C57BL/6J mice (Charles River Laboratory, Saint Germain sur l’Arbresle, France) purchased by Enterosys S.A. were housed in specific pathogen-free conditions and in a controlled environment (room temperature of 22 ± 2 °C, 12 h daylight cycle) with free access to food and water. After one week of acclimatization, mice were randomly assigned to experimental groups and fed a high-fat diet (HFD) containing fat 45% Kcal (Research Diet #D12451i, New Brunswick, NJ, USA) for 12 weeks (n = 8–10 per group). Treatment started concomitantly with the introduction of the HFD and consisted of daily oral gavage of 2 × 10^8^ cells of pasteurized *A. muciniphila* Muc^T^ in a volume of 180 μL of PBS containing 2.5% glycerol during the 12 weeks of HFD treatment. The batch of *A. muciniphila* Muc^T^ used in this study are fulfilling the criteria of novel food and has been cultured in and pasteurized as previously described [17,18]. The oral gavage was performed each day at the end of the light phase (end of afternoon). The vehicle was composed of PBS containing 2.5% glycerol. Prior to the beginning and at the end of the protocol, glycemia and insulinemia were assessed on blood samples collected from the tip of the tail vein after 6-h fasting. HOMA-index was calculated as follow: insulinemia (mU/L) x glycemia(mmol/L)/22.5. Body weight and food intake were assessed weekly.

### 2.3 Tissues sampling

After 12 weeks of treatment and in fed conditions, the animals were weighed, glycemia was measured and blood samples collected from the tip of the tail vein for further analysis. After exsanguination, mice were killed by cervical dislocation. Tissues (brown adipose tissue, epididymal adipose tissue, subcutaneous adipose tissue, visceral adipose tissue, proximal colon) were precisely dissected and weighed. All mouse experiments were approved by and performed in accordance with the guidelines of the local ethics committee (under ethical protocol APAFIS#11167-2018090712278926). The duodenum and colon segments and hypothalamus were also collected for the measurement of isotonic contraction and NO release described below.

### 2.4 Oral glucose tolerance test

At week 11, 6h-fasted mice were orally loaded with glucose (2 g/kg of body weight), around 18h after the last oral administration of vehicle or treatment. Glycemia was measured at −30, 0 (time of oral glucose loading), +15, +30, +45, +60, +90 and + 120 min with a gluco-meter (Accu-Chek Active, Roche) on blood samples collected from the tip of the tail vein. Blood was also collected from the tail vein at −30 and + 15 min with heparinized capillaries (≈60 μl) (sampling of two capillaries per mouse). The blood was centrifuged at 13000 g during 5 min at 4 °C. At least 15 μL of plasma was stored in a 0.5 ml tube at −80 °C until further analyses.

### 2.5 Isotonic contraction

After dissection, duodenum segments were washed and incubated in oxygenated Krebs-Ringer solution for 30 min at 37 °C, attached to the isotonic transducer (MLT7006 Isotonic Transducer, Hugo Basile, Comerio, Italy), and immersed in an organ bath of the same medium maintained at 37 °C. The load applied to the lever was 1 g (10 mN). Isotonic contractions were recorded on Labchart software (AD Instruments) following the transducer displacement. After attaching the intestinal segments, contractions were recorded for 15 min. The basal contractions were presented as average of amplitude and frequency of contraction.

### *2.6* *Ex vivo* hypothalamic and enteric real-time NO measurement

After dissection, duodenum and hypothalamus fragments were washed in Krebs–Ringer bicarbonate/glucose buffer (pH 7.4) in an atmosphere of 95% O_2_–5% CO_2_ and then immersed in Eppendorf tubes containing 400 μL of the same medium. After a 10 min recovery period, the spontaneous nitric oxide (NO) release was measured at 37 °C for 10 min by using a NO-specific amperometric probe (ISO–NOPF, 100 μm diameter, 5 mm length, World Precision Instruments, Aston Stevenage, UK) implanted directly into the tissue.

### 2.7 Data analysis

The data are expressed as the mean ± SEM. Differences between the experimental groups were assessed where appropriate using by one- or two-way ANOVA, followed by post-hoc Bonferroni's test. Data were analyzed using GraphPad Prism version 8.00 for Windows (GraphPad Software, San Diego, CA, USA). The results were considered statistically significant at *p* < 0.05.

## 3 Results

### 3.1 Pasteurized *A. muciniphila* decreases body weight gain

**Fig. 1:**
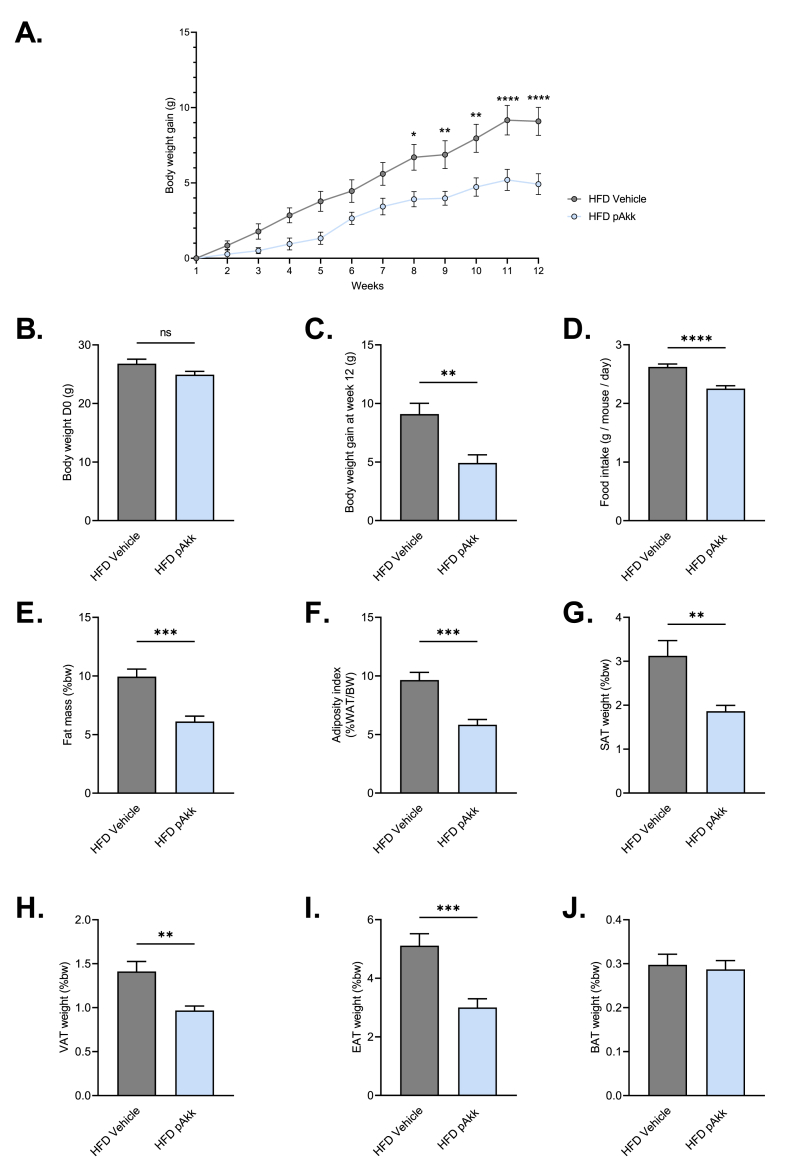
**Oral administration of pasteurized *A. muciniphila* improves body weight during a HFD45%.** Effects of an oral administration of vehicle or pasteurized *A. muciniphila* on **(A)** body weight gain evolution over time, *p < 0.05, **p < 0.01, ****p < 0.0001 *vs* HFD Vehicle, **(B)** body weight at the beginning of the protocol (D0), no significant difference was observed, **(C)** body weight gain at week 12 of treatment, **p < 0.01 *vs* HFD Vehicle, **(D)** food intake, ****p < 0.0001 *vs* HFD Vehicle, **(E)** Total fat mass ***p < 0.001 *vs* HFD Vehicle, **(F)** Adipose tissue index, ***p < 0.001 *vs* HFD Vehicle, **(G)** Subcutaneous adipose tissue (SAT), **p < 0.01 *vs* HFD Vehicle, **(H)** Visceral adipose tissue (VAT), **p < 0.01 *vs* HFD Vehicle, **(I)** Epididymal adipose tissue (EAT), ***p < 0.001 *vs* HFD Vehicle, **(J)** Brown adipose tissue (BAT), no significant difference was observed. The associated p-values obtained using an unpaired *t*-test. n = 8–10 per group.

We have started to explore the impact of pasteurized *A. muciniphila* on mice fed a high fat 45% (HFD), a model of diabetic mice with duodenal hypermotility. Compared to vehicle-treated HFD mice, pasteurized *A. muciniphila* treated present a significant reduced body weight gain reaching about 40% (Fig. 1A–C). This is associated with a significant decrease of food intake (Fig. 1D, HFD vehicle: 2.62 ± 0.04 *vs* HFD Akk: 2.25 ± 0.04 g/mouse/day), a decrease of fat mass (Fig. 1E, HFD vehicle: 9.94 ± 0.64 *vs* HFD Akk: 6.11 ± 0.45% of body weight) with a specific impact on adiposity (Fig. 1F, HFD vehicle: 9.65 ± 0.65 *vs* HFD Akk: 5.83 ± 0.45% of body weight) and white adipose tissue weights (Fig. 1G–I, Subcutaneous adipose tissue (SAT), HFD vehicle: 3.12 ± 0.34 *vs* HFD Akk: 1.86 ± 0.13% of body weight; Visceral adipose tissue (VAT), HFD vehicle: 1.41 ± 0.11 *vs* HFD Akk: 0.96 ± 0.05% of body weight; Epididymal adipose tissue (EAT), HFD vehicle: 5.11 ± 0.40 *vs* HFD Akk: 3.00 ± 0.29% of body weight), but no change in brown adipose tissue weight (Fig. 1J).

### 3.2 Pasteurized *A. muciniphila* improves glucose metabolism in diabetic mice

**Fig. 2:**
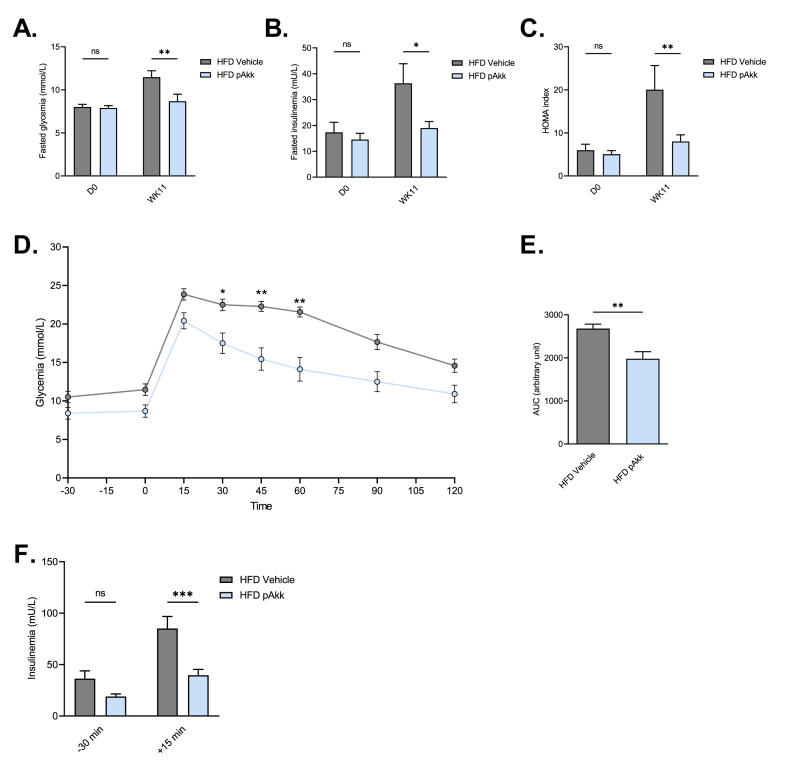
**Oral administration of pasteurized *A. muciniphila* improves glucose homeostasis during a HFD45%.** Effects of an oral administration of vehicle or pasteurized *A. muciniphila* on **(A)** fasted glycemia at the beginning (D0) and the end of the protocol (WK11), **p < 0.01 *vs* HFD Vehicle, **(B)** fasted insulinemia at the beginning (D0) and the end of the protocol (WK11), *p < 0.05 *vs* HFD Vehicle, **(C)** HOMA IR index calculated as insulinemia (mU/L) x glycemia (mmol/L)/22,5 at the beginning (D0) and the end of the protocol (WK11), **p < 0.01 *vs* HFD Vehicle, **(D)** Oral glucose tolerance test (OGTT) glycemia evolution before and after an oral load of glucose (2 g/kg of body weight), *p < 0.05, **p < 0.01 *vs* HFD Vehicle, **(E)** the average area under the curve (AUC) of the glycemia measured during the OGTT, **p < 0.01 *vs* HFD Vehicle, **(F)** Insulinemia before and 15 min after an oral load of glucose, ***p < 0.001 *vs* HFD Vehicle. The associated p values were obtained using a 2-way ANOVA followed by Bonferroni's post-hoc test, except for panel E using an unpaired *t*-test. n = 8–10 per group.

We found that pasteurized *A. muciniphila* treatment in HFD mice improves fasted glycemia (Fig. 2A, HFD vehicle: 11.47 ± 0.74 *vs* HFD Akk: 8.68 ± 0.80 mmol/L, Supplemental F. 1A), fasted insulinemia (Fig. 2B, HFD vehicle: 36.31 ± 7.55 *vs* HFD Akk: 18.97 ± 2.55 mU/L, Supplemental F. 1B), HOMA index (Fig. 2C, HFD vehicle: 20.05 ± 5.60 *vs* HFD Akk: 8.00 ± 1.54, Supplemental F. 1C), glucose tolerance (Fig. 2D, **E**) and insulin release in response to oral glucose (Fig. 2F).

### 3.3 Pasteurized *A. muciniphila* targets the “ENS/smooth muscle cells” couple

**Fig. 3:**
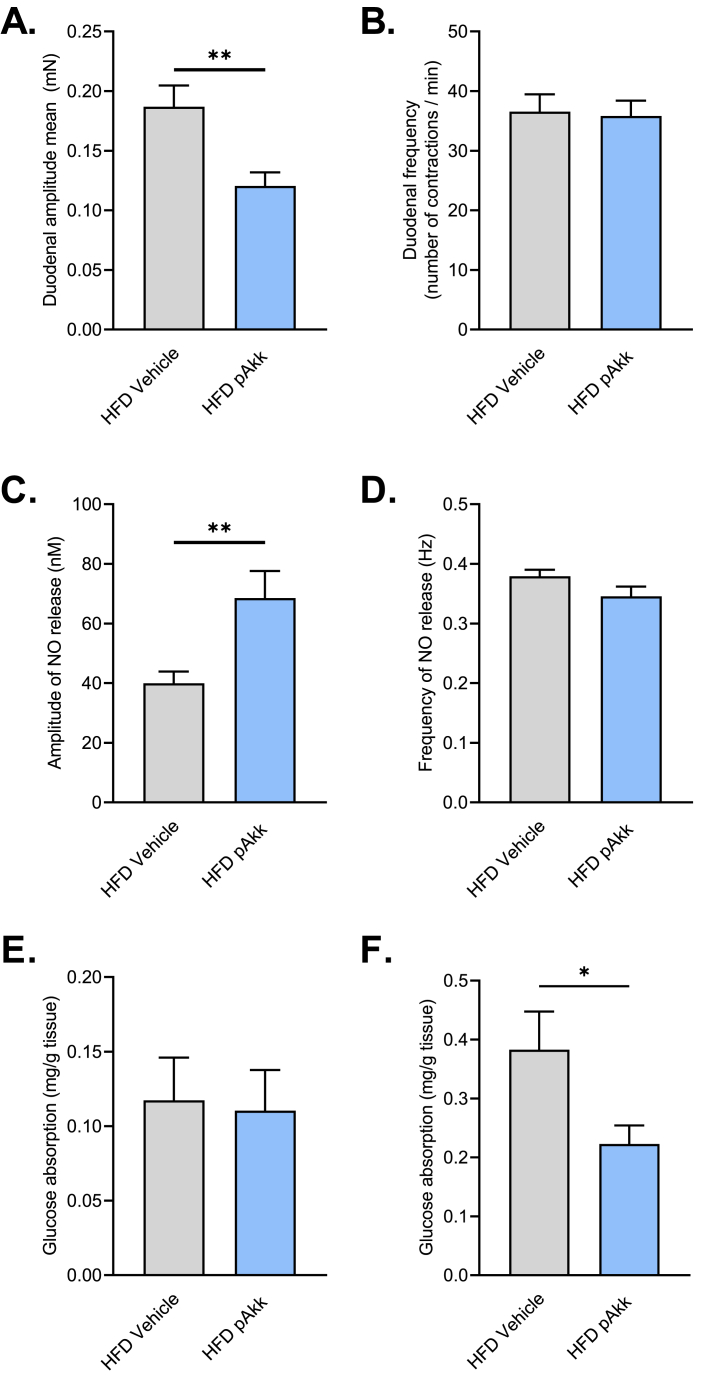
**Oral administration of pasteurized *A. muciniphila* modulates gut motility and decreases glucose absorption during a HFD45%.** Effects of an oral administration of vehicle or pasteurized *A. muciniphila* on **(A)***Ex vivo* measurement of duodenal mechanical contraction amplitude, **p < 0.01 *vs* HFD Vehicle, **(B)***Ex vivo* measurement of duodenal mechanical contraction frequency, no significant difference was observed, **(C)***Ex vivo* measurement of duodenal nitric oxide (NO) release amplitude during 10 min, **p < 0.01 *vs* HFD Vehicle, **(D)***Ex vivo* measurement of duodenal nitric oxide (NO) release frequency during 10 min, no significant difference was observed, **(E)***Ex vivo* glucose absorption in duodenal everted sacs, no significant difference was observed, **(F)***Ex vivo* glucose absorption in jejunal everted sacs, *p < 0.05 *vs* HFD Vehicle. The associated p values were obtained using an unpaired *t*-test. n = 8–10 per group.

We have previously shown that a decrease of duodenal hypermotility improves diabetic states. Here, we discovered that HFD mice treated with pasteurized *A. muciniphila* present a significant decrease of duodenal amplitude of contraction (Fig. 3A, HFD vehicle: 0.18 ± 0.01 *vs* HFD Akk: 0.12 ± 0.01 mN) without any change in the contraction frequency (Fig. 3B). This phenotype is correlated with an increase of the amplitude of NO spikes release from the duodenum (Fig. 3C, HFD vehicle: 36.96 ± 3.91 *vs* HFD Akk: 68.52 ± 9.07 nM), but not the frequency (Fig. 3D) and a significant decrease in glucose absorption in the proximal jejunum, where the majority of glucose is absorbed (Fig. 3F, HFD vehicle: 0.38 ± 0.06 *vs* HFD Akk: 0.22 ± 0.03 mg/g of tissue). No modification of the motility of the colon is observed between the 2 experimental groups (Supplemental F. 2A, B).

### 3.4 Pasteurized *A. muciniphila* restores the GBA axis during T2D

**Fig. 4:**
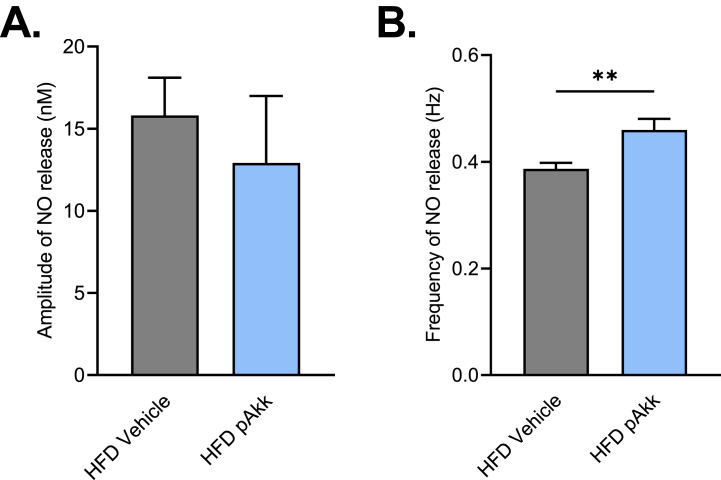
**Oral administration of pasteurized *A. muciniphila* restores the GBA during a HFD45%.** Effects of an oral administration of vehicle or pasteurized *A. muciniphila* on **(A)***Ex vivo* measurement of hypothalamic nitric oxide (NO) release amplitude during 10 min, no significant difference was observed, **(B)***Ex vivo* measurement of hypothalamic nitric oxide (NO) release frequency during 10 min, **p < 0.01 *vs* HFD Vehicle. The associated p values were obtained using an unpaired *t*-test. n = 9–10 per group.

The “gut contraction to hypothalamus to peripheral tissue” axis requires NO as a hypothalamic messenger. We observed that modification of gut motility in response to pasteurized *A. muciniphila* treatment in HFD mice significantly increases the frequency of release of hypothalamic NO (HFD vehicle: 0.38 ± 0.01 *vs* HFD Akk: 0.45 ± 0.02 Hz), without affecting the amplitude (Fig. 4A–B).

## 4 Discussion

Both live and pasteurized *A. muciniphila* have shown positive effects on cardiometabolic risk factors in several obesogenic and diabetic mice models as well as in humans with metabolic syndrome [13,14]. Several mechanisms of action have been proposed in order to explain its positive cardiometabolic effects. Among them, we can mention the role of the protein Amuc_1100 that binds to toll like receptor 2 (TLR2) thereby improving the gut barrier and reducing inflammation [15]. Other potential new mechanisms of action are under investigations in view of deciphering the beneficial effect of pasteurized *A. muciniphila* on glucose homeostasis [19]. Among these, it has recently been shown that supraphysiological doses of a protein called P9 and secreted by live *A. muciniphila* increases GLP-1 secretion [20,21]. However, this effect is absent in physiological conditions and obviously when *A. muciniphila* is not alive (i.e., pasteurized). In this study we tested the hypothesis that the beneficial effects of pasteurized *A. muciniphila* on glucose homeostasis and body weight gain could be linked to an improvement of the gut-brain-axis.

In the literature, HFD providing 60% of energy as lipids is a classical model to induce obesity, hyperglycemia, and insulin resistance in mice. The beneficial effects of pasteurized *A. muciniphila* in this model have been unequivocally demonstrated [15,16]. However, whether pasteurized *A. muciniphila* is efficient in other models such as HFD providing 45% of energy as lipids is unknown. We used a HFD45% model because this model allows us to investigate the altered enteric neurons activity in the proximal part of the intestine that characterizes diabetes. Indeed, investigating intestinal hyper-contractility is not technically measurable in a diet providing 60% of lipids due to important ectopic fat deposits in insulin sensitive tissues and in the gastro-intestinal tract. Then, the first step of our work was to test whether pasteurized *A. muciniphila* can replicate its beneficial effects on cardiometabolic disorders in an HFD-mice model providing 45% Kcal as lipids. In this study, we confirm that a daily oral administration of pasteurized *A. muciniphila* during 12 weeks in mice fed with HFD45% reduced body weight gain, decreased hyperglycemia and hyperinsulinemia in fasted condition and during an OGTT. This improvement of glucose homeostasis without a compensatory hyperinsulinemia is of great interest to delay/prevent pancreas exhaustion during the time course of the insulin-resistance progression [22]. Moreover, a significant decrease of the highest glucose levels (T30 – T60) after a bolus of glucose can have long term positive effect. Indeed, it is well established that postprandial hyperglycemia contributes significantly to the development of cardiovascular complications and correlating with increased incidence of cardiovascular events and mortality [23]. A recent 10-year post-trial follow-up study confirmed the importance of a low post-prandial glycemia. The study included 243 patients from the DIANA (DIAbetes and diffuse coronary Narrowing) study. They demonstrated that early improvement of post-prandial glucose significantly reduced major adverse cardiac events and unplanned coronary revascularization during the post-trial 10-year period in people with impaired glucose tolerance or newly-diagnosed T2D [24].

As mentioned earlier, several mechanisms were proposed to explain the positive effects of pasteurized *A. muciniphila* on hyperglycemia [12]. Briefly, *A. muciniphila* reduces metabolic endotoxemia by improving the gut barrier function and subsequently reducing low-grade inflammation and insulin-resistance. These effects are partly explaining by the interaction of a specific membrane protein (Amuc_1100) with TLR-2 [15] but also *via* specific bioactive lipids including short chain fatty acids and lipids related to the endocannabinoid system [25]. Recent research identified that targeting the ENS could be another mechanism playing a crucial role in the improvement glucose homeostasis [1]. Regarding the impact on *A. muciniphila* in the GBA, we may not rule out that the secretion of nano-sized extracellular vesicles (EVs) could occur. However, since we are dealing with a dead bacterium, although EVs have the great capacity to cross the mucus layers to reach the circulation, we do believe that this mechanism is not involved. Having said that, Ashrafian et al. [26] have shown that *A. muciniphila* and its EVs may improve glucose homeostasis of HFD mice. Whether the EVs could have a direct impact on glycemia by acting on peripheral tissue do not exclude a potential central effect *via* the GBA axis. How *A. muciniphila* and its EVs could modulate the GBA axis? In fact, oral administration of *A. muciniphila* and its EVs in rodents modulate the serotonin levels in the colon and in the hippocampus [27] demonstrating the new mode of communication between intestinal *A. muciniphila* and the brain. Altogether, these data suggest a link between gut microbiota and ENS dysfunction associated with serotonin metabolism during HFD [28] and is of crucial importance for the discovery of novel therapeutic targets.

Here, we have shown that pasteurized *A. muciniphila* can counteract the deleterious effect of a HFD on duodenal contraction amplitudes, NO release and glucose absorption in the proximal intestinal segment. The effect of pasteurized *A. muciniphila* on intestinal glucose absorption has already been suggested by Depommier et al. [16]. Indeed, they observed that pasteurized *A. muciniphila* reduced the expression of several carbohydrate transporters (GLUT2, GLUT5 and SGLT1) suggesting that pasteurized *A. muciniphila* lowered glucose and fructose absorption in the jejunum. However, whether pasteurized *A. muciniphila* decreases glucose absorption *in vivo* has never been investigated. Here we discovered that pasteurized *A. muciniphila* reduces duodenum contractility, increases NO release and decrease glucose absorption in the jejunum.

The development of obesity and insulin resistance in HFD-fed mice is in part attributed to GLP-1 resistance which reduces enteric activation and NO production [29]. The living form of *A. muciniphila* is able to produce short chain fatty acids (SCFA – acetate and propionate) and stimulate the production of specific bioactive lipids from the endocannabinoids system to stimulate the secretion of GLP-1 and GLP-2, contributing to regulation of glucose metabolism [12]. It is interesting to note that in the present study we found that the pasteurized form of the bacteria modulates ENS and NO production likely without affecting the ability to produce those specific molecules since the bacteria is dead and do not produce any SCFA or bioactive lipids. However, we may not rule out that the protein Amuc_1100 expressed on the membrane of pasteurized *A. muciniphila* contributes in part to the effect of the pasteurized form of the bacteria on the regulation of glycemia through the ENS and NO production.

Beside the effect of the bacteria at the gut level, we aimed to identify if the brain, mainly the hypothalamus, integrates these gut-derived signals, to improve glucose homeostasis [30]. We discovered that the oral administration of pasteurized *A. muciniphila* increases NO release amplitude in the hypothalamus which is correlated with a decrease of duodenal hypercontractility and glucose absorption at the proximal part of the intestine, corroborating a GBA action of the bacteria. These data confirmed the link between the couple “ENS/smooth muscle cells” and the hypothalamic NO release in diabetic mice. Indeed, the decrease of duodenal contractions leads to an increase of hypothalamic NO release, thus demonstrating that intestinal motility controls hypothalamic activity [8,10]. Finally, this coordinate mechanism impacts the overall glucose homeostasis as hyperglycemia and hyperinsulinemia are improved in our study. Nevertheless, one limitation of our study is the lack of direct evidence demonstrating the causal relationship between metabolic syndrome and NO release in the hypothalamus of mice administered with pasteurized *A. muciniphila.* Indeed, we have measured hypothalamic NO release in *ex vivo* condition. However, we have previously shown that the stimulation of intestinal cells by intragastric bioactive peptides (i.e. galanin) [10] or glucose [3] generates a quick release of hypothalamic NO measured with amperometric probe directly implanted in the brain of anesthetized mice. The measurement of hypothalamic NO with the same method could be used in pasteurized *A. muciniphila* treated mice compared to control. Another possibility would be to use inhibitors of NOS enzymes directly perfused in the brain of mice *via* an intracerebroventricular catheter in order to verify if the effects on metabolic functions of pasteurized *A. muciniphila* requires this NO signaling pathway.

In conclusion, our results show that oral administration of pasteurized *A. muciniphila* is also highly efficient to improve several metabolic parameters in mice fed with a HFD providing 45% of energy as lipids. Moreover, we discovered that pasteurized *A. muciniphila* improves blood glucose homeostasis (after an OGTT and a fasting period) through a modulation of the ENS, upper intestinal contractility together with a change in hypothalamic NO release. Our results are of importance and show that targeting the GBA can be a promising approach to prevent or treat insulin-resistance and T2D development. By using a specific paraprobiotic such as pasteurized *A. muciniphila*, we have shown that lowering hypercontractility and eventually glucose absorption in the upper part of the gastro-intestinal tract is a potential target. Further investigations are needed to decipher the full mechanism of action of the bacteria and whether it is *via* the ENS and GBA or *via* peptides or hormones or enterosynes that are also able to impact this regulation of glucose homeostasis.

## Author contribution statement

Anne Abot: Conceived and designed the experiments; Performed the experiments; Analyzed and interpreted the data; Wrote the paper.

Amandine Brochot, Patrice D. Cani: Conceived and designed the experiments; Analyzed and interpreted the data; Wrote the paper.

Nicolas Pomié: Gwendoline Astre: Performed the experiments; Analyzed and interpreted the data.

Céline Druart: Conceived and designed the experiments; Analyzed and interpreted the data.

Willem M. de Vos: Conceived and designed the experiments; Wrote the paper.

Claude Knauf: Analyzed and interpreted the data; Contributed reagents, materials, analysis tools or data; Wrote the paper.

## Funding statement

Patrice D. Cani was supported by 10.13039/501100002661Fonds De La Recherche Scientifique - FNRS {T.0030.21}, FRFS-WELBIO {WELBIO-CR-2022A-02}, ARC {ARC19/24-096} and La Caixa fundation (Barcenola, Spain) - Grant NeuroGut. Willem M. de Vos was supported by 10.13039/100017334Soehngen Institute of Anaerobic Microbiology (SIAM) Gravitation {024.002.002}, the 2008 Spinoza award of the Netherlands Organization of Scientific Research.

## Data availability statement

Data will be made available on request.

## Ethic statement

All mouse experiments were approved by and performed in accordance with the guidelines of the local ethics committee (under ethical protocol APAFIS#11167-2018090712278926).

## Declaration of competing interest

The authors declare that they have no known competing financial interests or personal relationships that could have appeared to influence the work reported in this paper.
