# Supplementary figures and images for: Pasteurized Akkermansia muciniphila improves glucose metabolism is linked with increased hypothalamic nitric oxide release
Source: Heliyon. 2023 Jul 13;9(7):e18196. doi: 10.1016/j.heliyon.2023.e18196 (PMC10368821; doi:10.1016/j.heliyon.2023.e18196)

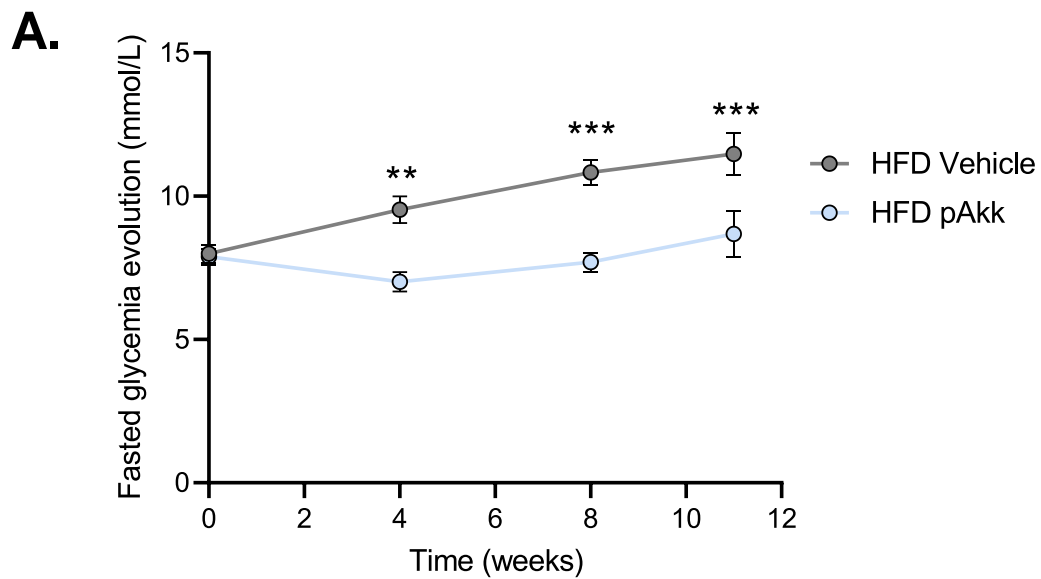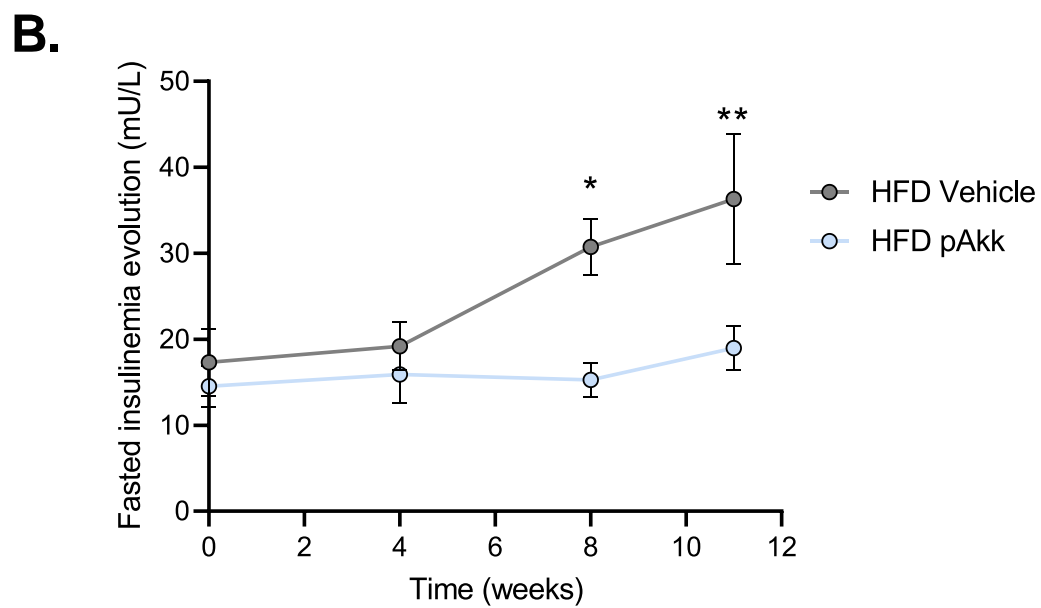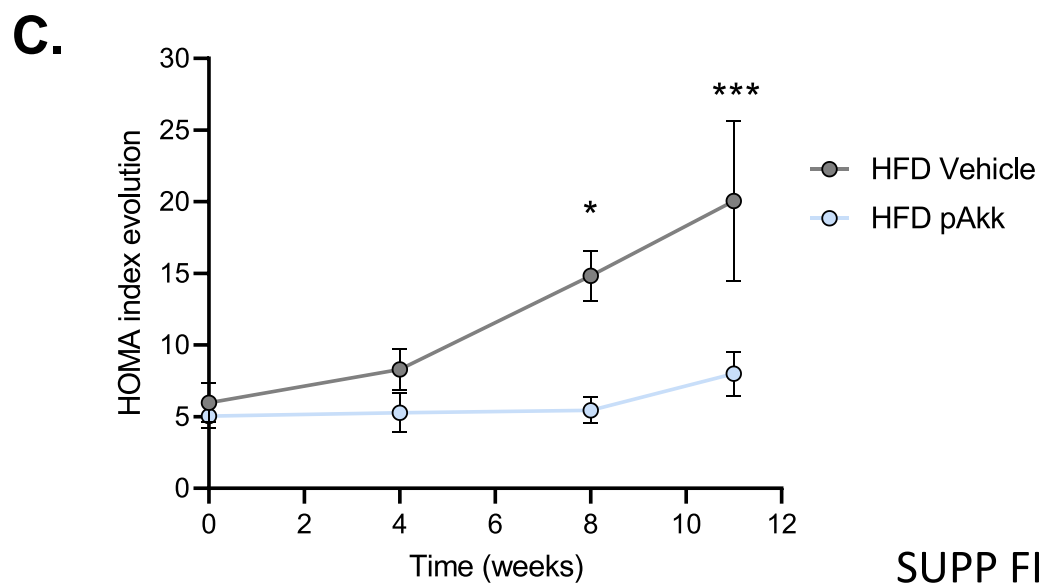

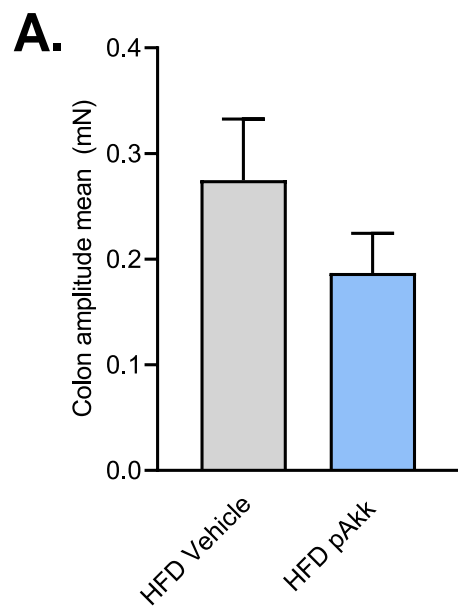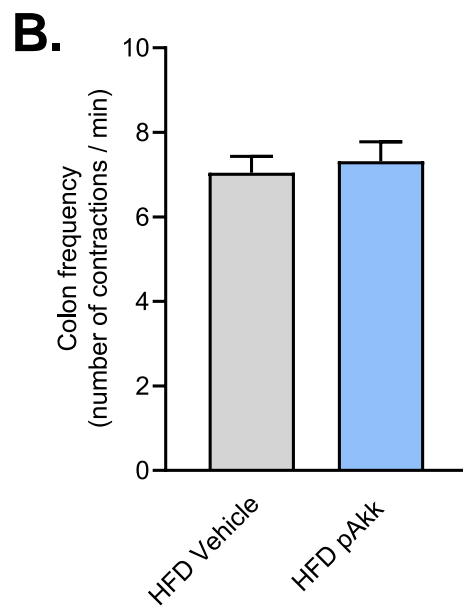

Supplement: Multimedia component 1 [file mmc1.pdf]
